# Supplementary material for: Evaluation of a health information exchange system for microcephaly case-finding — New York City, 2013—2015
Source: PLoS One. 2020 Aug 17;15(8):e0237392. doi: 10.1371/journal.pone.0237392 (PMC7430720; doi:10.1371/journal.pone.0237392)
Supplement: Attachment — Submitted filename: Response to Reviewers.docx [file pone.0237392.s001.docx]

Dear Academic Editor:

Thank you for inviting us to submit a revised version of our manuscript entitled “Evaluation of a health information exchange system for microcephaly case-finding — New York City, 2013—2015" to PLOS ONE. Following is a rebuttal letter that responds to each point raised by the academic editor and reviewers.

**Review Comments to the Author**

*Academic Editor:*

The Authors are expected to address the comments by all Reviewers. In particular, please provide more details on how the data were queried (Reviewers #1 and #3), how missing data was handled (Reviewer #2), differences between the two reporting systems, the analysis and state the study objective clearly (Reviewer #3).

***Response: Thank you for the constructive feedback. Below the authors have responded and addressed the comments made by all reviewers with particular emphasis on providing more details on how the data were queried (Reviewers #1 and #3), how missing data was handled (Reviewer #2), differences between the two reporting systems, the analysis and study objective (Reviewer #3).***

*Reviewer #1:*

**Comment 1.** Thank you for this opportunity to review this paper. As someone who has worked at the federal, state, regional, and organization level on HIE, I found this very interesting. This manuscript was very well written, easy to read, and easy to follow.

***Response:* *Thank you – we are pleased you found the study to be interesting and clearly written.***

My minor comments are as follows:

**Comment 2.** This may have more relevance in other surveillance situations, not just microcephaly. This should be stated.

***Response: Thank you for the comment. We completely agree. HIE systems present important opportunities to advance public health surveillance, in ways that can increase timeliness and completeness of public health surveillance. The literature has linked HIE systems to improvements in child and adolescent immunization status, timeliness of notifiable disease reporting, reductions in duplicative diagnostic testing and identification of drug seeking behaviors, and improved identification of high utilizing vulnerable patients returning within 72 hours of initial emergency department discharge. The Introduction of the manuscript now references the literature citing these examples where HIE systems have been used to enhance disease surveillance practices.***

***In the discussion, the authors do describe scenarios where HIEs could be used to support newborn microcephaly surveillance. Additional language has now been added to clarify that these applications could similarly supplement traditional approaches to disease surveillance and facilitate surveillance for other conditions of public health interest, including emerging health threats.***

**Comment 3.** The methods sections was light. There needs to be more around how the data were queried.

***Response: The authors have added more language to the methods around how the data were queried. The methods now further describe the HIE database, the approach taken to query and identify records meeting criteria, the data elements on suspected cases identified by HIE that were extracted for use in the match analysis.***

**Comment 4.** Discussion and Conclusion. More details around the case that was missed (L146)

***Response: We agree with the reviewer that this case is perplexing. As we now indicate in our manuscript, we found no explanation for the missed case, but hypothesize that the diagnostic code was recorded in a secondary field in the EMR that was picked up by the HIE but not by the reporting hospital’s query in the NYSRCR study.***

*Reviewer #2:*

**Comment 1.** The study aims at evaluation of Health Information Exchange (HIE) data at three Bronx hospitals in New York City for surveillance and detection of microcephaly cases diagnosed at birth during Jan 1, 2013–Dec 31, 2015 before Zika virus introduction in North America. The use of HIE data along with other data sources for surveillance is a known practice. Methodologically the paper has shown little novelty. The domain, the application and results are interesting, although the sensitivity 58.21%, seems low. I’m also curious to know how the authors deal with missing data in the HIE and the chart reviews. Also, I suggest the authors to discuss potential biases in this study.

***Response: In this analysis, the authors did not return to the charts the HIE missed to uncover the reasons why these cases were missed. However, the authors suspect it may be due to a lack of consistent and quality EMR documentation. The authors have now addressed this point in the limitations section of the Discussion.***

Minor comments:

**Comment 2.** Line 61: “these rapid and active surveillance components has” should be “these rapid and active surveillance components have”

***Response: We agree. The phrase has been revised and now reads, “these rapid and active surveillance components have…”.***

**Comment 3.** Line 88: “was excluded from analysis” should be “was excluded from the analysis”

***Response: Revision made. The sentence now reads, “one suspected case born to a non-NYC resident mother was excluded from the analysis”.***

**Comment 4.** Line 103: “One of 4 cases meet the microcephaly case definition” should be “One of 4 cases meets the microcephaly case definition”

***Response: Suggested change made.***

**Comment 5.** Line 166: “First, detection of” should be “First, the detection of”

***Response: Change made. The sentence now reads, “First, the detection of cases using HIE data was limited to microcephaly diagnoses queried using a single ICD-CM-9 or ICD-CM-10 code on day of birth or during the first two days after birth”.***

*Reviewer #3:* The authors present an interesting analysis although the manuscript is vague and does not provide enough detail to properly evaluate the work.

***Response: Thank you. We are pleased you found the analysis interesting. We have revised the manuscript to address the comments you outline below.***

Major comments:

**Comment 1.** The objective of the study isn't well defined. Based on lines 73-74, the objective was to estimate how well the suspected case definition performs in terms of specificity and sensitivity?

***Response: We have revised the introduction to clarify the objective and rationale for our approach. The manuscript now reads, “******The objective of this analysis was to determine*** ***the extent to which querying of HIE data could replicate the yield obtained by the NYSRCR study through more labor-intensive and costly approaches, involving direct inquiry of hospital records, querying administrative discharge data, and chart abstraction. The sensitivity, specificity, positive and negative predictive values of querying HIE data for identifying confirmed cases of microcephaly were estimated.******”***

**Comment 2.** The differences between the two reporting systems needs to be more clearly described. For example, routine reporting relies on a congenital malformation registry that is based on hospital reports (using ICD 10 codes) whereas the rapid reports are based on the same codes but are more frequently obtained from the same hospitals (I'm assuming that the ICD codes for Zika congenital syndrome has been updated in the routine reporting as well).

***Response: Thank you for the comment. In the manuscript, the authors describe 3 reporting systems: 1) routine reporting, 2) direct inquiry of hospitals followed by medical chart review, and 3) querying of HIE data. Routine reporting (#1) is introduced in the Introduction as relying on hospitals and providers to report individual cases to congenital malformations registries in accordance with state public health laws and regulations. However, the authors did not attempt to compare HIE to routine CMR reporting, which may have led to confusion by the reviewer. Surveillance of birth defects in New York conducted by the New York Congenital Malformations Registry receives reports from hospitals on major birth defects in infants and children diagnosed before the age of 2 years. Thus, routine CMR reporting is not helpful for outbreak detection or case-finding.***

***The analysis described in this manuscript evaluated the extent to which querying of HIE data (#2)*** ***could replicate the yield obtained in the NYSRCR study through direct inquiry of hospital records (#3). Using the same criteria (diagnosis codes) and case definition as those used for the NYSRCR study, the authors evaluated the use of querying HIE data to replace direct inquiry of hospitals or enhance direct inquiry of hospitals by confirming cases identified through both systems and identifying additional cases not captured through hospital inquiry. HIE can replace or complement direct inquiry depending on resources available. We have revised the Methods to clarify the reporting systems being compared, and Discussion and Conclusion to more directly address these points and reduce confusion.***

**Comment 3.** It also sounds like it was a capture-recapture approach that was taken for the study? If so, please further describe the approach as it will help the reader understand what has been done and the objective of the study. Nothing is mentioned at all about the analysis - how were the data obtained (in what format), what about basic characteristics of the suspected cases (any information on the mothers, when were they born, etc). Record linkage through what exactly? how were estimates of sensitivity, specificity, etc were estimated - using what type of regression? Sensitivity analysis based on the specificity and sensitivity of the case definitions should also be factored into the estimates.

***Response: Thank you for the comment. The authors did not design this as a capture-recapture study. The design of the analysis was a criterion-related validation study where direct inquiry of hospitals followed by medical chart review/abstraction was the*** ***“gold standard” model. The study design for this analysis was premised on the assumption that direct inquiry would capture 100% of cases.***

***The revised manuscript expands on the methods of the analysis to address the concerns of the reviewer. Cases identified using the same microcephaly diagnostic codes born to NYC resident mothers were matched for three birth hospitals covered by both data sources using birth hospital name, date of birth, and medical record number. The select birth hospitals in the Bronx had cases in the NYSRCR that were also in the HIE system. Records were linked and matched using a deterministic approach. Cases were classified as a match if the two records agreed on all identifiers and a nonmatch if the two records disagreed on any of the identifiers. The definitions of sensitivity, specificity, negative predictive value, and positive predictive value are described.***

***No regression analysis or adjustments to sensitivity and specificity estimates were made. This analysis did not attempt to evaluate the microcephaly case definitions developed by CDC and the National Birth Defects Prevention Network (NBDPN). Our goal was not to see whether the expanded list of ICD-10-CM codes used in Zika Birth Defects Surveillance (ZBDS) captures the entire population of children with congenital microcephaly. Rather, this analysis attempted to apply the same microcephaly diagnostic codes (ICD-9-CM or ICD-10-CM) to two data sources (captured via querying HIE data or through direct inquiry of hospital records) to see if the yield was comparable. A limitation we mention in the discussion is that the HIE diagnosis was required to occur in within 2 days of birth but could have been made at any time during the newborn stay for the NYSRCR study.***

**Comment 4.** Case definitions should be provided as supplemental information.

***Response: Thank you for the comment. We have clarified our descriptions of suspected and confirmed cases and now explain that NYSRCR records were defined as non-cases (that is, not confirmed) if they had been misclassified (e.g., macrocephaly, microcephalus) or because both a physician diagnosis and anthropometric information needed to accurately classify head circumference percentile were missing. In addition, we have referred readers to the below reference [8] for further information on NBDPN case definitions which is publicly available to interested readers:***

***National Birth Defects Prevention Network (NBDPN). NBDPN abstractor’s instructions. Houston, TX: National Birth Defects Prevention Network; 2016.*** [***http://www.nbdpn.org/docs/NBDPN_Case_Definition-SurveillanceMicrocephaly2016Apr11.pdf***](about:blank)

**Comment 5.** In terms of data availability, what data are available exactly and how (contact corresponding author)?

***Response: Thank you for the inquiry. The authors have made available all the data required to replicate the analysis described in this manuscript. The data can be found in Table 1. For readers interested in the data used in the New York State Retrospective Chart Review study [Graham et al. 2017] and information abstracted from charts by trained clinicals, we would refer readers to contact the corresponding authors Deborah J. Fox (***[***deb.fox@health.nyc.gov***](about:blank)***) and Krishika A. Graham (***[***kgraham1@health.nyc.gov***](about:blank)***) for data requests.***
