# Supplementary material for: Evaluation of a health information exchange system for microcephaly case-finding — New York City, 2013—2015
Source: PLoS One. 2020 Aug 17;15(8):e0237392. doi: 10.1371/journal.pone.0237392 (PMC7430720; doi:10.1371/journal.pone.0237392)
Supplement: Attachment — Submitted filename: Response to Reviewers.docx [file pone.0237392.s002.docx]

Dear Academic Editor:

Thank you for inviting us to submit a revised version of our manuscript entitled “Evaluation of a health information exchange system for microcephaly case-finding — New York City, 2013—2015" to PLOS ONE. Following is a rebuttal letter that responds to each point raised by the academic editor and reviewers.

**Review Comments to the Author**

*Academic Editor:*

The Authors are expected to address the comments by Reviewers #3. In additional to these comments, please address:

1. Abstract, please add the 95% Cis for sensitivity, PPV and NPV.

***Response: Thank you for the feedback. The authors have added the 95% CI for sensitivity, PPV, and NPV to the abstract. Additionally, the authors have responded and addressed the comments made by all Reviewer #3 below.***

*Review Comments to the Author:*

Please use the space provided to explain your answers to the questions above. You may also include additional comments for the author, including concerns about dual publication, research ethics, or publication ethics. (Please upload your review as an attachment if it exceeds 20,000 characters)

Reviewer #2: The comments and concerns have been promptly addressed. The paper is acceptable and contributes to the field.

***Response:* *Thank you – we are pleased you found that the revised manuscript acceptable for publication and a contribution to the field.***

Reviewer #3: The authors did an impressive job at thoroughly addressing all of the comments. The manuscript is clear and the objectives and approach of the study are well described. I have only a few minor comments that need addressing:

***Response:* *Thank you for the constructive feedback. We have addressed and responded to the minor comments that need addressing.***

**Comment 1.** In the methods section, it should be stated how the 95% CIs were generated (for Table 2) as well as using a Kappa statistic.

***Response: That authors have now added a statement to specify that corresponding Clopper-Pearson exact 95% confidence intervals were estimated around the parameters generated in Table 2. Standard logit confidence intervals were initially used for the predictive values but have been modified to reflect more conservative Clopper-Pearson exact 95% confidence intervals. A sentence has also been added to the methods section to indicate that a kappa statistic was calculated to measure correspondence between the two systems. All statistical analyses were performed using SAS 9.4 (SAS Institute Inc, Cary, North Carolina) and can be replicated. The authors state this clearly in the methods section.***

**Comment 2.** Table 2 - don't include (2013-2015) in column heading

***Response:***  ***The authors have removed ‘(2013-2015)’ from the column heading in Table 2.***

**Comment 3.** Line 256 - Preferable to not start a sentence with And

***Response: The authors have revised the sentence. The sentence does not start with the word ‘And’ but now reads, “In fact, despite the increasing adoption and implementation of EMR systems, there has been a lack of standardization in design and structure, as well as in adoption of documentation workflows.”***

**Comment 4.** Discussion - An important limitation of using HIE, as mentioned, is the poor sensitivity. The alternative approach (starting line 195), would seem the most reasonable/main way forward, as HIE would need to be supplemented with chart reviews. I would suggest that this should be reworded in terms of alternative approach.

***Response:* *In the discussion, the authors do describe scenarios where HIEs could be used to support newborn microcephaly surveillance. The authors introduce the alternative approach of using HIE data to identify cases requiring confirmatory chart review in scenarios where direct inquiry is warranted. The authors have now reworked the Discussion to describe this alternative approach more clearly. The authors are more explicit about how adding automated HIE reporting to a request and review system could improve efficiency. For systems that do rely on hospital inquiry and confirmatory chart review, cases identified by both systems could bypass human review so that limited resources could be devoted to investigating cases that are only picked up by one source.***

**Comment 5.** Discussion - limitation #2 - this is an important point for recommendations. There needs to be better standardization of reporting across EMRs, which is likely the root of the problem. I would make an explicit recommendation here.

***Response: We completely agree. We have made a recommendation for better standardization of reporting across EMRs to the Discussion. The manuscript now reads, “These variations call for better standardization of reporting and HIE practices across EMR systems. Continued effort to develop standards for EMR design that will produce clearer documentation of the clinical workflow and requirements for data capture could lessen these challenges and improve how EMR systems can be used for public health surveillance and assist health departments”.***
